# ANZAED practice and training standards for dietitians providing eating disorder treatment

**DOI:** 10.1186/s40337-020-00334-z

**Published:** 2020-12-15

**Authors:** Gabriella Heruc, Susan Hart, Garalynne Stiles, Kate Fleming, Anjanette Casey, Fiona Sutherland, Shane Jeffrey, Michelle Roberton, Kim Hurst

**Affiliations:** 1 Executive Committee, Australia & New Zealand Academy for Eating Disorders, Sydney, Australia; 2 School of Medicine, Western Sydney University, Campbelltown, Australia; 3 Eating Disorder Service, Northern Sydney Local Health District, Sydney, Australia; 4 Nutrition and Dietetics, St Vincent’s Hospital, Darlinghurst, Australia; 5 The Boden Collaboration of Obesity, Nutrition, Exercise and Eating Disorders, The University of Sydney, Sydney, Australia; 6 School of Sport, Exercise and Nutrition, College of Health, Massey University, Auckland, New Zealand; 7 The Swan Centre, Perth, Australia; 8 Centre for Psychotherapy, Hunter New England Local Health District, Newcastle, Australia; 9 The Mindful Dietitian, Melbourne, Australia; 10 River Oak Health, Brisbane, Australia; 11 Royal Brisbane and Women’s Hospital, Brisbane, Australia; 12 Victorian Centre of Excellence in Eating Disorders, Parkville, Australia; 13 Eating Disorder Service, Robina Private Hospital, Robina, Australia; 14 Griffith University, Gold Coast, Australia

**Keywords:** Dietetic, Dietitian, Eating disorder, Nutrition, Practice standards, Training, Treatment

## Abstract

**Introduction:**

Dietitians involved in eating disorder treatment are viewed as important members of the multidisciplinary team. However, the skills and knowledge that they require are not well characterised. Therefore, as part of a broader project to identify the key principles and clinical practice and training standards for mental health professionals and dietitians providing eating disorder treatment, the Australia & New Zealand Academy for Eating Disorders (ANZAED) sought to identify the key practice and training standards specific to dietitians. An expert working group of dietitians was convened to draft the initial dietetic standards. After expert review, feedback on the revised standards was then provided by 100 health professionals working within the eating disorder sector. This was collated into a revised version made available online for public consultation, with input received from treatment professionals, professional bodies and consumer/carer organisations.

**Recommendations:**

Dietitians providing treatment to individuals with an eating disorder should follow ANZAED’s general principles and clinical practice standards for mental health professionals and dietitians. In addition, they should also be competent in the present eating disorder-specific standards based around the core dietetic skills of screening, professional responsibility, assessment, nutrition diagnosis, intervention, monitoring and evaluation.

**Conclusions:**

These standards provide guidance on the expectations of dietetic management to ensure the safe and effective treatment of individuals with an eating disorder. Implications for professional development content and training providers are discussed, as well as the importance of clinical supervision to support professional self-care and evidence-informed and safe practice for individuals with an eating disorder.

## Plain English summary

Dietitians play an important role in eating disorder treatment, however the skills and knowledge they require to provide safe and effective treatment is not well defined. This paper aimed to describe the clinical practice and training standards for dietitians providing eating disorder treatment. The Australia & New Zealand Academy for Eating Disorders undertook extensive consultation to establish agreement around what constitutes best dietetic practice in the treatment of individuals with an eating disorder. The resulting standards were based around the core dietetic skills of screening, professional responsibility, assessment, nutrition diagnosis, intervention, monitoring and evaluation, and also outlined the expectations for training and supervision.

## Introduction

The role of the dietitian in providing eating disorder treatment as part of the multidisciplinary team has been widely recognised [1–3]. Dietitians play a pivotal role in helping individuals with eating disorders and their families understand the interaction between food, nutrition and well-being, as well as supporting eating behaviours that align with their treatment and recovery goals. Eating disorders have high morbidity and mortality rates [4], and failure to provide early intervention is associated with a longer duration and severity of illness, serious physical health consequences and a higher risk of mortality including risk of suicide [5]. However, morbidity and mortality in individuals with an eating disorder can be improved with effective treatment [5]. Nutritional care contributes to improving quality of life and morbidity and mortality rates in general dietetics [6], but the dietitian’s role in eating disorder treatment is less clear and the specifics of dietetic practice are not well defined [7].

Although current clinical practice guidelines recommend dietetic assessment, education and intervention as part of the multidisciplinary treatment of eating disorders [1–3], there is a lack of detailed guidance for dietitians regarding what outpatient dietetic treatment for eating disorders should encompass [8]. Moreover, there is an absence of internationally accepted dietetic models of care within the eating disorders field, which may increase the likelihood of inconsistent interventions [9] and limit clarity regarding the role of the dietitian, both within the dietetic profession [10] and more broadly for other health professionals [11]. Dietetic scope of practice in the treatment of eating disorders is diverse, but at a minimum should include a tailored nutrition care process that: corrects nutritional deficiencies and promotes optimal nutrition status; addresses the role of eating and adequate nutrition in physical and mental well-being; and provides nutrition education to challenge inaccurate beliefs about food [2, 7, 12, 13]. Having clearly defined practice standards for dietitians will ensure effective, safe and timely care for individuals with an eating disorder in addition to consistent treatment approaches.

The role of dietitians in eating disorder treatment also appears to be affected by the adequacy of dietitian training with dietitians feeling that dietetic training is inadequate preparation for practice in eating disorders [14, 15]. Communication and nutrition counselling in particular are often insufficiently addressed at university, resulting in a gap in skillset for most dietitians [16, 17]. Thus, dietitians providing eating disorder treatment often seek further clinical experience, post graduate training and professional supervision [14]. To support ethical and effective dietetic care in eating disorder treatment, clarification is required on the knowledge and skills that training programs need to address.

The current clinical practice and training standards aim to describe the role of the dietitian in eating disorder treatment and provide a roadmap for dietitians to provide effective and safe care. This document builds on the Australia & New Zealand Academy for Eating Disorders (ANZAED) general principles and practice and trainings standards for all clinicians providing eating disorder treatment concurrently published in the Journal of Eating Disorders [18]. Based around the core dietetic skills of screening, professional responsibility, assessment, nutrition diagnosis, intervention, monitoring and evaluation, it details the knowledge and skills that dietitians require to competently manage and treat individuals with an eating disorder. It also describes the expectations and content required to be addressed in training programs that provide education on the therapeutic knowledge and skills outlined in these practice standards. Consistent and standardised dietetic practice may not only enhance the legitimacy and credibility of dietitians as part of the multidisciplinary team, but may also lead to improvements in clinical care for individuals with eating disorders.

## Methods of dietetic-specific practice and training standards development

ANZAED established an expert working group of eight dietitians with representatives from Australia and New Zealand, each with between 12 and 30 years of clinical experience in eating disorder treatment. This was part of a broader project to develop general clinical practice and training standards [18], as well as standards specific to mental health professionals [19] and dietitians providing eating disorder treatment.

The working group initially developed draft dietetic-specific standards based on published evidence and clinical experience, which were reviewed and refined by ANZAED’s Executive Committee and expert advisors. As part of a combined clinical practice and training standards document, the next draft version was then presented for public face-to-face consultation without anonymity at the ANZAED 2019 Conference in Adelaide, with feedback received and incorporated from eating disorder professionals (*n* = 100). Following this, online consultation from the broader public was sought, with feedback received from international expert advisors, professional bodies (various disciplines) and consumer and carer groups with comments reviewed and integrated by the working groups. The final version was also reviewed by the National Eating Disorder Collaboration Steering Committee and again by ANZAED’s Executive Committee. Consensus was reached through discussion by the authors and there was no identified conflict of interest. The resulting dietetic-specific clinical practice standards are detailed below and in Table 1.

**Table 1:**
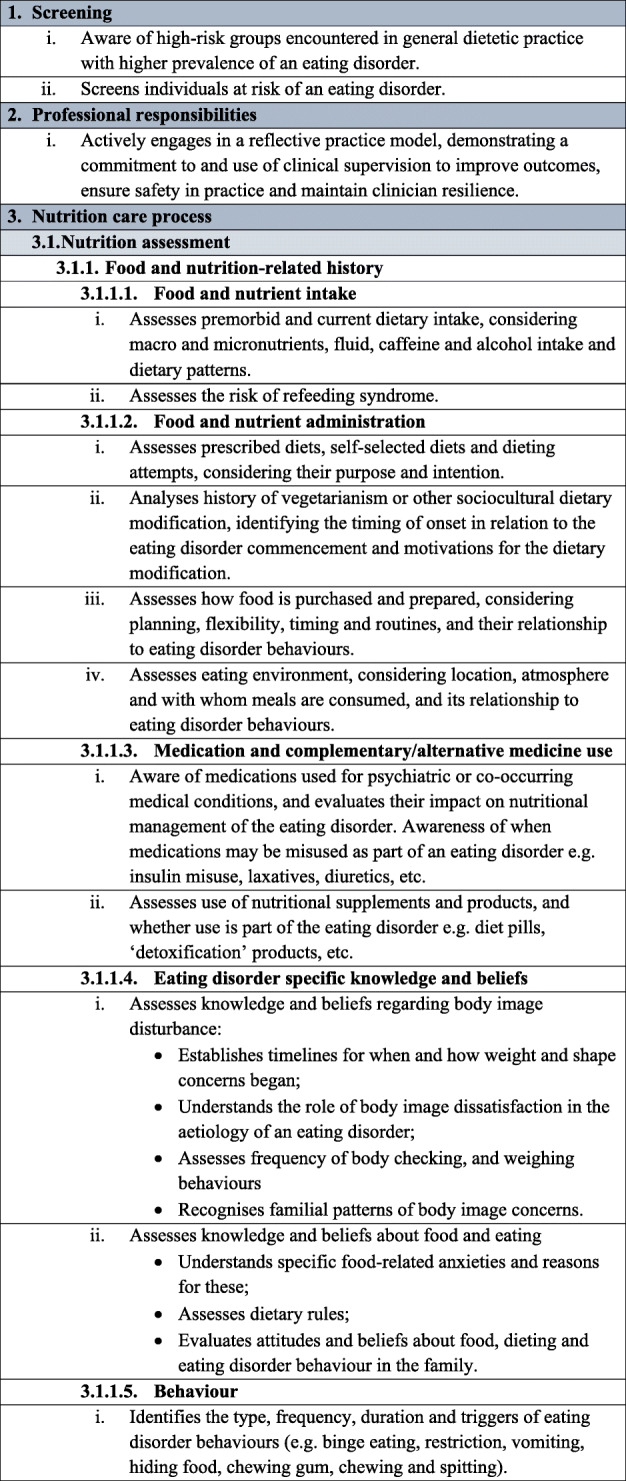

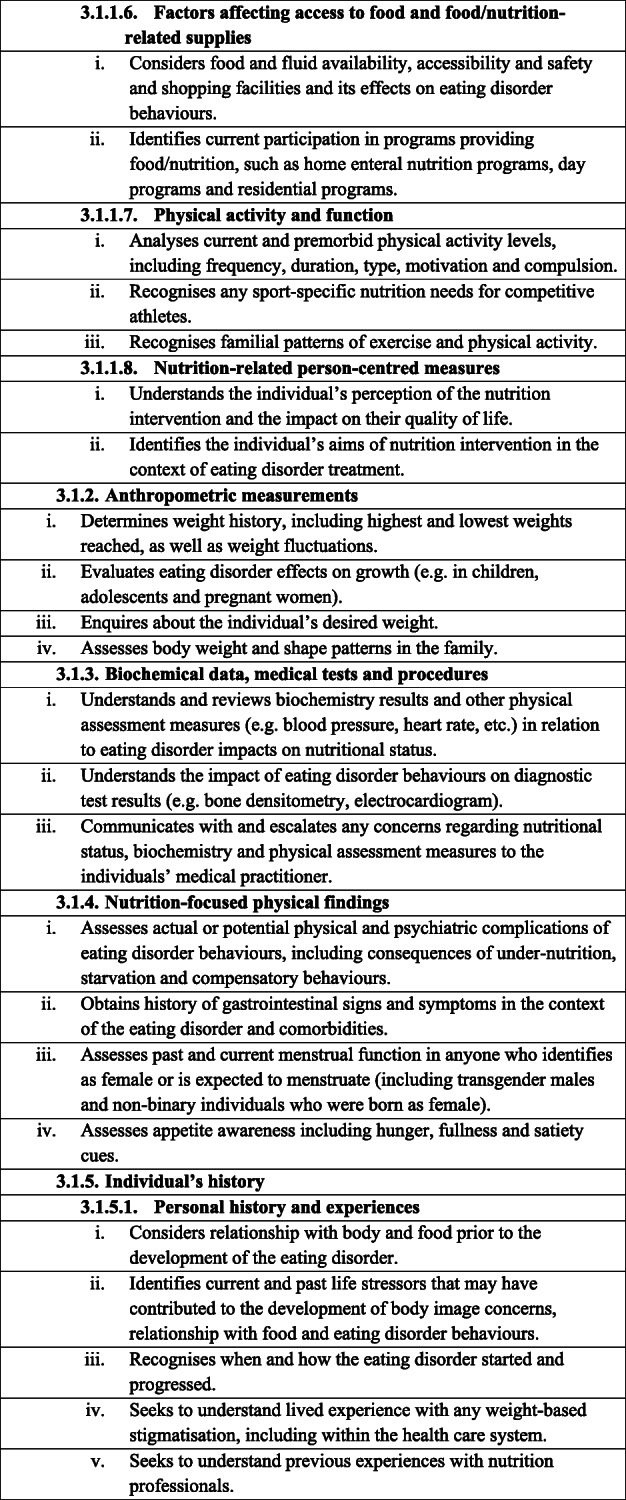

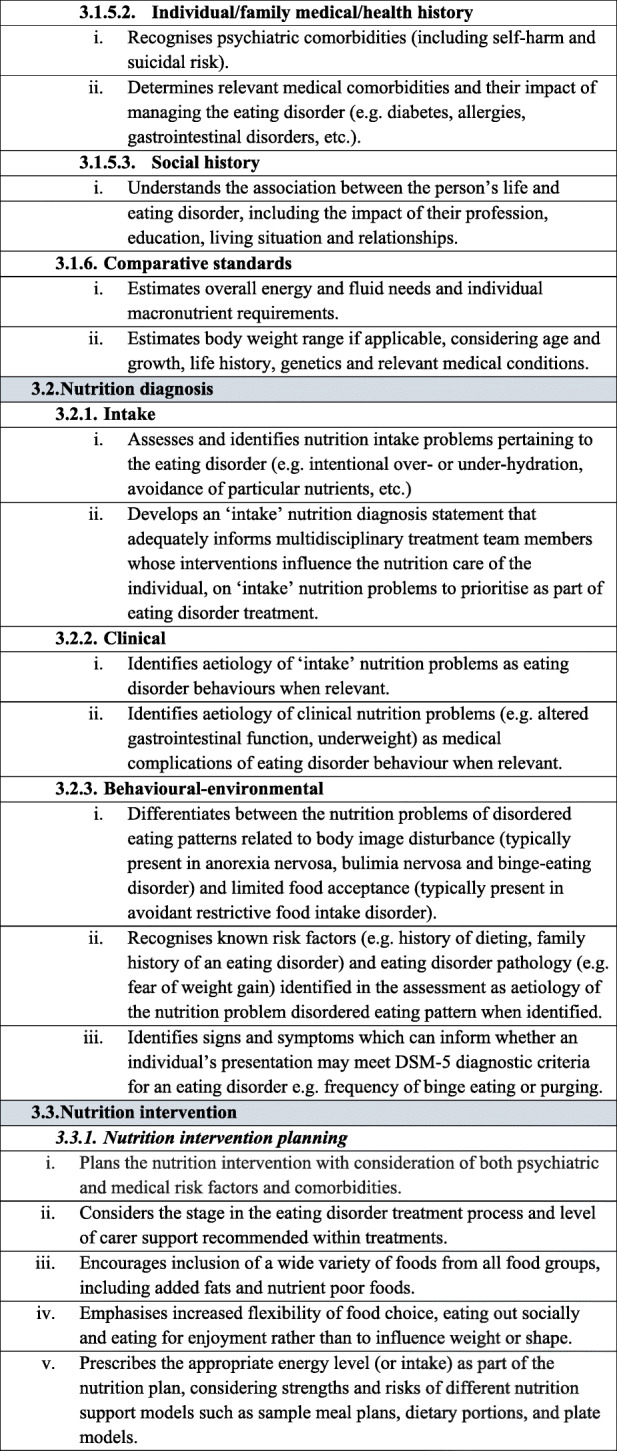

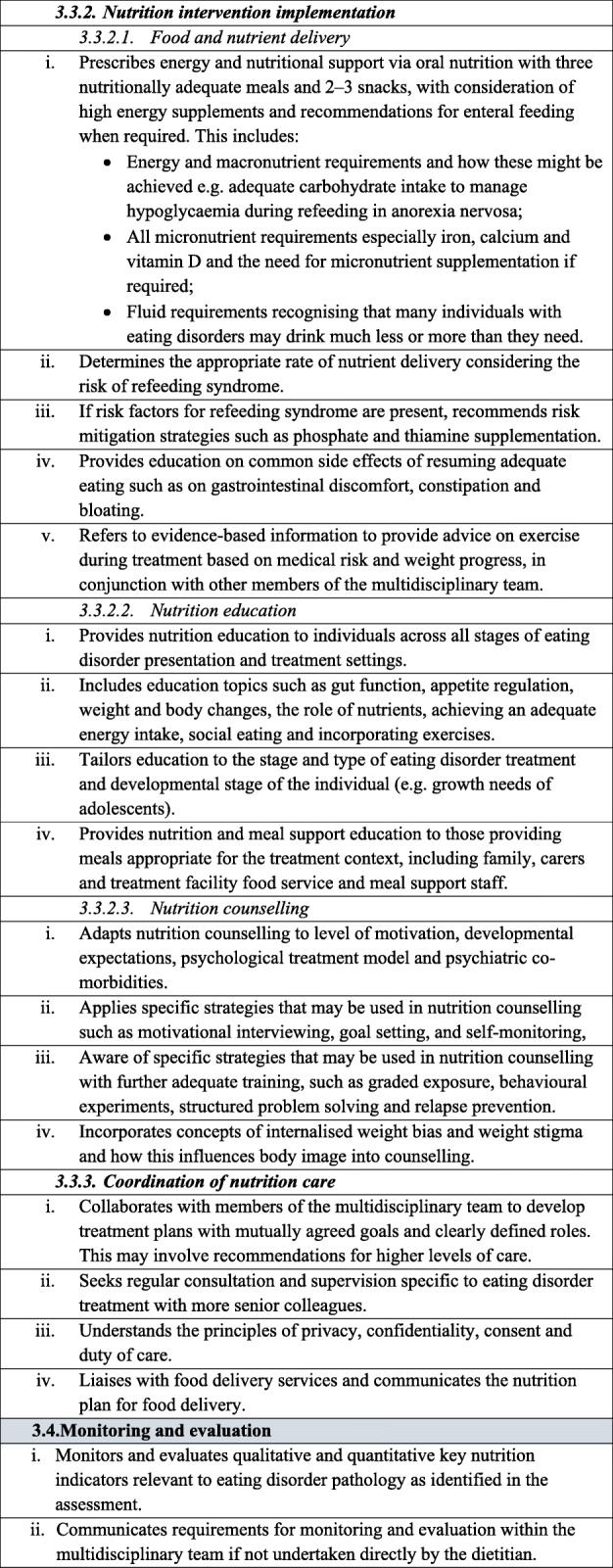
Dietetic-specific clinical practice and training standards. The table below describes in detail the dietetic-specific practice and training standards that were summarised in the Recommendations section. It outlines the specific practice points recommended that dietitians be taught, understand and utilise to ensure safe and effective eating disorder treatment

## Recommendations

### Dietetic-specific clinical practice standards

It is recommended that dietitians providing treatment to individuals with an eating disorder follow the broad principles and practice standards outlined in The Australia & New Zealand Academy for Eating Disorders treatment principles and general clinical practice and training standards for mental health professionals and dietitians [18]. In addition, to provide safe and effective treatment, it is recommended that dietitians treating individuals with an eating disorder are also competent in the dietetic-specific clinical practice standards summarised below and detailed in Table 1**.** General dietetic skills are assumed, in line with dietetic competencies set out by relevant dietetic registering bodies, and are not mentioned here.

#### Screening

The prevalence of eating disorder presentations in general dietetic practice remains unknown. However, all dietitians have an important role in early identification and the screening of high-risk individuals using evidence-based tools, such as the SCOFF [20], and BEDS-7 [21]. For many individuals with an eating disorder there are no, or few, obvious signs of ill-health. Without timely and appropriate screening and assessment, an opportunity for detection of symptoms may be missed [22, 23].

Individuals who should be screened for an eating disorder may present to a dietitian to discuss their dietary concerns without specifically seeking treatment or support for an eating disorder. Individuals might belong to groups where there is a higher prevalence of eating disorders, including those: (a) at higher [24] or lower body weights [25]; (b) with recent rapid weight loss or gain [26]; (c) presenting for weight management with concomitant significant concerns about appearance and/or repeated efforts to change body shape (pursuing weight loss, or gain) [27]; (d) following a self-imposed (e.g. gluten-free, food allergy−/intolerance-related, vegan) [27, 28] or medically prescribed restrictive diet (e.g. due to type 1 diabetes, coeliac disease [29–31] or low ‘FODMAP’ diet due to irritable bowel syndrome [32]); (e) post-bariatric surgery [33]; (f) presenting with unspecified gastrointestinal symptoms such as constipation or abdominal pain [34, 35], or with a diagnosis of irritable bowel syndrome [32]; (g) presenting with physical symptoms or electrolyte disturbance that could be attributed to starvation/malnutrition or purging behaviour [36]; (h) being a restrained eater [37], or restricting energy and nutritional intake [38]; (i) having a concurrent mental health concern [22, 39]; and (j) elite athletes who participate in individual sports or that require meeting a weight criterion (e.g. lightweight rowers, jockeys, martial arts practitioners, boxers, dancers, gymnasts) [40].

#### Professional responsibilities

Consistent recommendations are for dietetic intervention to be part of a multidisciplinary team intervention, and not an intervention to be delivered in isolation [2, 41].

Clinicians in eating disorder practice are also advised to have clinical supervision as a form of formal learning and reflective practice [18]. Although clinical supervision is not historically utilised in the dietetics profession [42], dietitians do highly value learning from mentors [14]. As seen in New Zealand [43], dietitians would likely benefit from more formal clinical supervision, with the aim of encouraging safe and competent practice [42]. More specifically, dietitians working in the mental health and eating disorders fields believe that supervision is required to help dietitians deal with challenging behaviours and relationships [42, 44]. The British Dietetic Association has introduced professional supervision to the dietetic profession, defining it as a “process of professional support and learning undertaken through a range of activities, which enables individuals to develop knowledge and competence, assume responsibility for their own practice and enhance service-user protection, quality and safety of care” [45]. Clinical dietetic supervision should use a model that is suited to the dietetics profession and learning style [46]. Clinical supervision has been reported by allied health professionals to be most effective when professional development was the focus of the supervision, the supervisor possessed the skills and attributes required to facilitate a constructive supervisory relationship and the workplace provided an environment that facilitated this relationship and professional development [46]. Furthermore, since dietetics training often does not have a strong counselling focus, it may be important for the supervisee and supervisor to undergo formal training to ensure competency of supervision skills [46].

#### Nutrition care process

The following has been developed in line with the Nutrition Care Process Terminology (NCPT) [47]. The purpose of NCPT is to provide an accurate and specific description of the services that nutrition and dietetics practitioners deliver. The aim is to achieve a common understanding of these services not only among nutrition and dietetics practitioners, but also outside the profession, including individuals with an eating disorder and other members of the multidisciplinary team [48]. Standardized terminology facilitates the clear description of nutrition care through the following four steps of the Nutrition Care Process: nutrition assessment, nutrition diagnosis, nutrition intervention, and nutrition monitoring and evaluation [49].

##### Nutrition assessment

All dietitians are trained to undertake a nutrition assessment. However, when obtaining an assessment of an individual with an eating disorder (or suspected eating disorder), additional questions and tailoring of the assessment is required. Factors across the assessment domains relevant to both physical and mental health need to be considered. Particular attention should be given to the individual’s beliefs about food, any dietary rules, dieting behaviour, food avoidance, attempts to reduce weight and weight history [2]. Additionally enquiring about and being aware of physical and psychological signs and symptoms of starvation such as preoccupation with food [50], hypothermia [50], bradycardia [51], postural hypotension [51], GI dysfunction [34], appetite disturbance [52], social isolation [50], depression [53] and hypoglycaemia [54] is useful. Dietitians specialising in eating disorder treatment should also ask about the frequency and triggers for eating disorder behaviours such as binge eating, and compensatory behaviours such as vomiting, laxative use, and excessive exercise [2]. It is important to be aware that eating disorders can occur in individuals of any age, gender or body size, cultural background and demographic [55].

##### Nutrition diagnoses

Dietitians working in the eating disorders field identify and manage specific nutrition problems and diagnoses resulting from the psychological and physical complications associated with an eating disorder (e.g. malnutrition) [2]. A nutrition diagnosis is different from a medical/psychiatric diagnosis, and while identifying eating disorder signs and symptoms is expected, providing a medical or psychiatric diagnosis is not within the dietitian’s scope of practice.

##### Nutrition intervention

The role of the dietitian is to identify, plan and implement appropriate nutrition interventions with the purpose of modifying nutrition-related health status, behaviours, knowledge and attitudes to achieve physical, psychological and nutritional recovery, as well as support behaviours and attitudes to best sustain an individual’s wellbeing [2]. This places the dietitian in an ideal role to contribute towards eating disorder recovery. A standardised nutrition intervention should involve prioritising the goals and expected outcomes, the development of an appropriate nutrition plan as required, establishment of interdisciplinary connections and the implementation, documentation and revision of the plan as required [2]. Wherever possible, the nutrition intervention should be collaboratively undertaken with the client.

*Nutrition intervention planning*. Eating disorders present both psychiatric and medical risk which needs to be considered in planning nutrition interventions. Given the dietary rigidity present in eating disorders, dietitians need to consider how their interventions either support or discourage eating flexibility and normalised eating patterns. In contrast to broad public healthy eating guidelines, nutrition interventions should be designed to minimise exclusion of any foods including those considered nutrient poor. Individuals with an eating disorder generally experience high levels of anxiety about eating, which may affect the individual’s readiness for change and tolerance of uncertainty. Dietitians need to tailor their treatments to support individuals towards recovery while providing safe and ethical nutrition interventions [7, 56, 57].

*Nutrition intervention implementation*

1. Food and nutrient delivery: Food and nutrient delivery are tailored to the individual’s nutrition needs, but the individual’s stage of recovery will help to inform the treatment plan. The treatment plan considers evidence about specific foods and nutrients for weight restoration, appetite regulation, lifecycle nutrition, the presence of co-occurring psychiatric and medical conditions that affect nutritional status, and stage of recovery. In an inpatient setting, dietitians may advise the multidisciplinary team on the most appropriate method of nutrient delivery such as oral feeding, nutrition supplements and/or nasogastric feeding [12, 57–59].
2. Nutrition education: Nutrition education is provided throughout treatment and across all treatment settings. This includes information regarding energy and nutrient needs [60], the impact of food and nutrients on physical and psychological wellbeing, effects of energy or nutritional deficiency, appetite cues and the relationship between dietary intake and exercise. Other topics that may be relevant for nutrition education include dietary iron and calcium requirements, family eating patterns, eating socially, shopping and cooking skills, metabolism and gastrointestinal function [59]. Education should be directed towards the person who is most responsible for making eating decisions and this may be the individual, family, or carer and is dependent on the psychological treatment model [56]. Education serves to support the individual and/or family in considering the potential benefits of incorporating sustainable change in eating patterns and behaviours to promote the recovery process. The specific timing and topics of education should be person-centred, varying according to the stage of change.
3. Nutrition counselling: Nutrition counselling is provided to individuals and families, and complements the psychological model used in therapy. Nutrition and dietetic counselling practices have been identified and include but are not limited to monitoring eating behaviour, beliefs and attitudes about food and health, rationale for food choice, factors affecting eating behaviour and nutritional status, factors affecting access to food, motivation and stages of change and addressing ambivalence and barriers to behaviour change [61]. There should be an awareness of the evidence-based psychological models used in eating disorder treatment (as outlined by Hilbert et al. [62]). Understanding and complementing a psychological model with dietetic care as part of the multidisciplinary team is different from the dietitian implementing the model, and while understanding the principles of models of care for eating disorders is expected, implementing a psychological treatment model is not considered to be within the dietitian’s scope of practice without significant additional mental health training and supervision.

*Coordination of nutrition care*. Due to the high level of multi- and interdisciplinary care that occurs in eating disorder treatment, coordinating nutrition care between professionals is particularly important. Eating disorder treatment necessarily includes traditionally dietetic tasks, such as weighing individuals and discussing food and eating. Defining clear roles, boundaries and communication pathways between professionals involved in care can help streamline effective care, minimise professional burnout and enhance client outcomes [44, 63].

##### Monitoring and evaluation

Throughout treatment, ongoing nutritional monitoring is required to evaluate outcomes of treatment and particularly change in eating disorder behaviour. Due to the focus on nutritional recovery in eating disorder treatment, other members of the treatment team will also likely have a role in ongoing monitoring and evaluation. It is important that treatment outcomes are evaluated both qualitatively (e.g. change in the individual’s perceived relationship with food) and quantitatively (e.g. change in nutritional intake). New and developing concerns need to be addressed with the individual and communicated to the rest of the treatment team as a lack of change in eating and eating disordered behaviour may indicate a need to review treatment.

### Dietetic-specific clinical training standards

Dietetic-specific clinical training should either partially or entirely address the dietetic-specific clinical practice standards outlined above and detailed in Table 1, depending on the duration and intensity of the training course. It is expected that training programs that educate dietitians working with individuals with eating disorders should identify which of the standards they address. The dietetic-specific clinical training standards use the NCPT. Currently, dietitians broadly agree that university training does not adequately prepare graduates for providing eating disorder nutrition care and additional training after graduation is often sought [10, 14, 15]. Thus, after basic university dietetic training, training to achieve competency in these practice standards may occur via multiple pathways, including formal (e.g. postgraduate coursework, workshops, conferences) and informal (e.g. reading, intuitive learning through experience) learning opportunities [14]. Organisations and individuals providing training in the dietetic management of eating disorders should be suitably qualified, recognised for their expertise in dietetic eating disorder treatment and have significant experience implementing eating disorder nutrition care.

## Implementation and future directions

This paper provides a comprehensive outline of the skills and knowledge that a dietitian requires to provide effective and safe eating disorder treatment. It also describes the training and supervision required by dietitians, with the practice standards forming the basis of the essential elements of training. With the growing recognition that basic dietetic training at university does not adequately prepare dietitians for working with individuals with eating disorders [10, 14, 15], these practice and training standards may guide the enhancement of undergraduate coursework on the skills and knowledge necessary for such work and the development of postgraduate learning pathways.

The role and scope of practice of the dietitian presented also clarifies the skills and knowledge that the dietitian contributes to the multidisciplinary treatment team. It increases the understanding of how dietetic intervention assists individuals to achieve recovery from an eating disorder and ensures accurate nutrition advice is delivered [11]. Furthermore, it builds an appreciation of the specialised perspective that each member of the multidisciplinary team brings to a coordinated treatment plan, creating a continuum of cohesive care for the individual with an eating disorder.

Documentation of the current practice and training standards meet a long-standing need of the dietetic community who deliver eating disorder treatment, detailing the minimum skills and knowledge in which a dietitian should be trained. However, a process for determining competence to provide dietetic treatment in the eating disorder field has not yet been characterised. The future incorporation of these practice standards into training processes and programs is an essential first-step to ensure future dietitians can be adequately educated and supported to work in the eating disorder field.

## Data Availability

Not applicable.
